# Reductive degradation of carbon tetrachloride using tree leaf polyphenol–iron complexes for groundwater remediation†

**DOI:** 10.1039/d5ra01391g

**Published:** 2025-07-04

**Authors:** Roselle Colastre Lasagas, Chenju Liang, Xuyen Thi Hong Luong, Florencio Ballesteros

**Affiliations:** a Department of Environmental Engineering, National Chung Hsing University 145 Xingda Road, South Dist. Taichung City 402202 Taiwan cliang@nchu.edu.tw xuyenluong@yahoo.com lasagas.roselle@gmail.com +886-4-22856610 +886-4-22856610; b Environmental Engineering Graduate Program, University of the Philippines Diliman Philippines; c Department of Chemical Engineering, University of the Philippines Diliman Philippines fcballesteros@up.edu.ph

## Abstract

Plant polyphenols, natural antioxidants, form complexes with iron minerals that enhance contaminant degradation *via* reductive processes. This study investigated the degradation of carbon tetrachloride (CT) using polyphenol–iron complexes synthesized from tree leaf extracts. Polyphenols were extracted from waste tree leaves, including *Ficus microcarpa*, *Terminalia neotaliala*, *Haematoxylon campechianum*, *Ficus septica*, *Mangifera indica*, and *Ficus religiosa*, with gallic acid identified as the predominant constituent. Among them, *Terminalia neotaliala* exhibited superior antioxidant capacity, reducing power, metal-chelating ability, and total phenolic content, making it the optimal choice for CT degradation experiments. Using the Taguchi method, optimal conditions for CT degradation were determined as pH 10, a leaf extract dose of 10 g L^−1^, and an Fe^2+^ concentration of 15 mM, with pH as the most influential factor. Under these conditions, CT degradation reached 99% in aqueous solution and 89% in field groundwater within 24 h. Detected intermediates included trichloromethane, dichloromethane, and chloromethane, with chloride ions as the final mineralization product. This study underscores the potential of tree leaf polyphenols, in combination with Fe^2+^, as a sustainable approach for groundwater remediation.

## Abstract 2

Tree leaf polyphenols, natural antioxidants, form complexes with iron minerals that enhance contaminant degradation *via* reductive processes.

## Graphical abstract

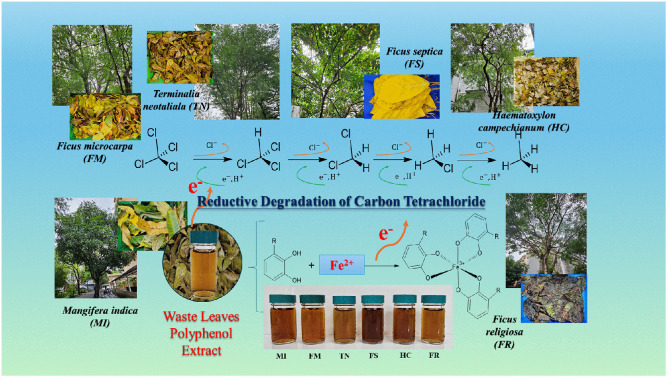

## 1. Introduction

Carbon tetrachloride (CT) is a synthetic chlorinated solvent that has been widely used in applications such as pesticides, chlorofluorocarbon production, aerosol propellants, dry cleaning, metal degreasing, and the manufacturing of rubber and paint.^1^ CT is highly toxic to humans and poses significant risks to aquatic ecosystems.^2^ In the event of a spill, CT, as a dense nonaqueous phase liquid (DNAPL), can infiltrate the ground and persist as a long-term source of groundwater contamination, potentially lasting for months or even years. Subsurface CT contamination can be addressed through air stripping, adsorption, or *in situ* chemical reduction (ISCR). Unlike air stripping and adsorption, which merely transfer contaminants from one medium to another, ISCR chemically transforms contaminants into non-toxic or less harmful compounds. Common reductants employed in ISCR include micron- or nano-scale zero valent iron (nZVI)^3^ and sodium dithionite.^4^ However, the application of these agents presents several challenges, including potential toxicity, the generation of hazardous by-products, and operational limitations. For instance, nZVI is typically synthesized using sodium borohydride, a process that may introduce secondary toxic pollutants. Moreover, production methods such as gas-phase reduction or electrochemical synthesis are energy-intensive and require specialized equipment. Once deployed, nZVI particles tend to aggregate rapidly, limiting their mobility and reactivity in subsurface environments.^3^ Similarly, the use of sodium dithionite can mobilize redox-sensitive elements and introduce excess sodium and sulfate into the aquifer, potentially affecting groundwater quality.^4^ More recently, ascorbic acid has shown promise as a reducing agent under alkaline conditions, effectively removing CT at pH 12.^5^ However, it is highly unstable in aqueous solutions due to rapid oxidation. These challenges have spurred interest in alternative reductants that are both effective and environmentally benign. Naturally occurring polyphenols have emerged as a promising class of reducing agents, offering a greener and potentially more sustainable approach to subsurface remediation.

Polyphenols are a class of phytochemicals present in all vegetative parts of plants and can also be persistent in plant debris after decomposition. Their key functional properties include scavenging free radicals, donating hydrogen atoms or electrons, and chelating metal ions.^6^ Polyphenols are categorized according to the number of phenol rings and structural elements that link them.^7^ There are four main categories of polyphenols: phenolic acids (hydrobenzoic acids and hydroxycinnamic acids), flavonoids, stilbenes, and lignans.^8^ Ho *et al.*^9^ demonstrated that tree leaves possess significant antioxidant activities and polyphenolic constituents. Specifically, as an example, the leaves of *Acer oliverianum* were found to contain a total phenolic content (TPC) of 311.7 ± 7.7 mg Gallic Acid Equivalent (GAE) per g sample, with phenolic acids and flavonoids being the predominant phytochemicals.

Polyphenols can chelate ferrous ion (Fe^2+^), which is another potent reducing agent that can be found in iron minerals. In the study of Wang & Liang,^10^ the combination of tea polyphenol and Fe^2+^ created an elevated reducing potential and achieved complete degradation of organochlorine pesticide. In addition, the Fe^2+^ and green tea complex was demonstrated to be capable of degrading various halogenated compounds, while Fe^2+^ or green tea alone were found to be less effective. The *ortho*-dihydroxyl groups such as catechol and gallol groups are responsible of the metal chelating ability of polyphenols. The formation of iron complexes promotes the release of H^+^ and the recycle of Fe^2+^/Fe^3+^, leading to an increase in the total dissolved iron in the solution. This accelerated reduction of ferric to ferrous ions might induce enhanced degradation of organic contaminants.^11–13^ The polyphenol–iron complex is pH dependent.^12^

Polyphenols are widely recognized for their antioxidant properties; however, under specific conditions, such as variations in pH, the presence of oxygen, and metal ions, they can also exhibit pro-oxidant behavior, leading to the generation of reactive oxygen species (ROS).^14^ The coordination chemistry between polyphenols and iron varies with pH: under acidic conditions, a monocomplex typically forms, consisting of a single polyphenol ligand bound to Fe^2+^ or Fe^3+^. At neutral and alkaline pH, biscomplexes (two ligands) and triscomplexes (three ligands), respectively, become more favorable.^15,16^ In the presence of oxygen, polyphenol groups such as catechol or gallate can rapidly oxidize Fe^2+^ to Fe^3+^, resulting in the formation of stable Fe^3+^–polyphenol complexes. During this redox process, the polyphenol ligand donates an electron to reduce Fe^3+^ back to Fe^2+^ while being oxidized to a semiquinone radical (SQ˙). Under acidic conditions, this semiquinone can further oxidize to quinone, concurrently reducing Fe^3+^ to Fe^2+^ or molecular oxygen to the superoxide radical (O_2_˙^−^). The superoxide can spontaneously dismutate to hydrogen peroxide (H_2_O_2_), which in turn reacts with Fe^2+^ in a Fenton reaction to produce highly reactive hydroxyl radicals (˙OH). These radicals are capable of degrading a wide range of organic contaminants. However, the overall reaction efficiency can be limited by the slow reduction of Fe^3+^ back to Fe^2+^, which constrains the generation of ˙OH and, consequently, the degradation of organic compounds.^17^ At neutral pH, the formation of biscomplexes may enable an alternative pathway, in which Fe^3+^ reacts with H_2_O_2_ to form high-valent iron species such as Fe^4+^, a potent oxidant. In contrast, under alkaline conditions, triscomplexes are more stable and exhibit antioxidant behavior. Their fully occupied coordination sites inhibit interactions with H_2_O_2_, preventing ROS generation. These triscomplexes can also chelate metal ions, reducing their availability for redox cycling and free radical formation. Furthermore, triscomplexes may facilitate the reductive degradation of CT *via* a controlled electron transfer mechanism, sustaining the Fe^3+^/Fe^2+^ redox cycle while minimizing undesired ROS production.^18^

This study aimed to investigate the degradation of CT utilizing waste tree leaf extract (TLE) as a source of polyphenols to form complex with Fe^2+^ additive in aqueous solution. The specific objectives were to: (1) determine the composition and characteristics of TLE; (2) optimize leaf dose, pH, and iron dose using Taguchi orthogonal array for the reductive degradation of CT in aqueous solution; (3) investigate the degradation by-products and reaction mechanism; (4) assess the removal of CT in field groundwater solution using the optimal conditions.

## 2. Materials and methods

### 2.1 Chemicals and materials

Linoleic acid (C_18_H_32_O_2_, ≥99%), ascorbic acid (C_6_H_8_O_6_, 99%), ferrozine (C_20_H_13_N_4_NaO_6_S_2_, 98%), gallic acid ((HO)_3_C_6_H_2_CO_2_H, 97.5%), Folin & Ciocalteu's phenol reagent (2 N), caffeic acid (C_9_H_8_O_4_, 98%), 4-hydrobenzoic acid (C_7_H_6_O_3_, 99%), tannic acid (C_76_H_52_O_46_), vanillic acid (C_8_H_8_O_4_, 97%), and magnesium chloride (MgCl_2_, ≥99%) were purchased from Sigma-Aldrich. Trichloroacetic acid (C_2_HCl_3_O_2_, 99.8%), citric acid (C_6_H_8_O_7_, 99.5%), sodium bicarbonate (NaHCO_3_, min. 99.7%), and potassium ferricyanide (K_3_(Fe(CN)_6_), 99%) were purchased from J.T. Baker. Iron chloride hexahydrate (FeCl_3_·6H_2_O, 98%) and iron chloride tetrahydrate (FeCl_2_·4H_2_O, 98%) were purchased from Alfa Aesar. Potassium phosphate dibasic (K_2_HPO_4_, 98%) was obtained from Nihon Shiyaku Industries Ltd. Potassium phosphate (KH_2_PO_4_, 99%) was purchased from Shimakyu Co., Ltd. Methanol (CH_3_OH, 99.9%) was obtained from Duksan. Potassium chloride (KCl, 99.5%), sodium carbonate (Na_2_CO_3_, 99.8%), and sodium chloride (NaCl, ≥99.5%) were purchased from Honeywell. Mixed anion standard – 7 components (fluorides, chlorides, nitrites, bromides, nitrates, sulphates, and phosphates) was purchased from CPAchem and calcium sulfate dihydrate (CaSO_4_·2H_2_O, 99%) was from Merck. Carbon tetrachloride (CCl_4_, min. 99.7%) was purchased from ALPS CHEM Co., Ltd while ferrous sulfate heptahydrate (FeSO_4_·7H_2_O, min. 98%) was purchased from Union Chemicals World Ltd.

The selection of tree species was guided by their availability on the campus of National Chung Hsing University (NCHU), their capacity to produce substantial leaf biomass, and their phytochemical profiles, particularly the abundance of polyphenols that are water-soluble. Water was selected as the extraction solvent due to its non-toxic nature and minimal risk of introducing additional contamination. The species evaluated for their phytochemical content and antioxidant potential included *Ficus microcarpa* (FM),^19^*Terminalia neotaliala* (TN),^20^*Haematoxylon campechianum* (HC),^21^*Mangifera indica* (MI),^22^*Ficus septica* (FS),^23^ and *Ficus religiosa* (FR).^24^ These trees are reported to be rich in polyphenols, particularly water-soluble phenolic acids, and possess notable antioxidant properties. MI, HC, and TN are deciduous species that shed all their leaves annually, producing significant biomass. FR is semi-deciduous, exhibiting partial and seasonal leaf drop. In contrast, FM and FS are non-deciduous, with FM having a broad, dense canopy and FS producing large leaves; both shed foliage gradually throughout the year. Groundwater was collected in a campus groundwater monitoring well (diameter of 4 in, a water depth of 15 m, and well screen positioned between 3.0 and 15.0 m) located at the Department of Environmental Engineering, NCHU. The deionized (DI) water used was generated using an Elga Micra Type II purification system.

### 2.2 Experimental procedures

The experimental flow chart is presented in Fig. S1 (ESI).† The first stage of the experiment was the characterization of waste tree leaves. There were six tree leaves that were considered and polyphenol compounds were extracted from these tree leaves in a form of filtrate extract using DI water as solvent. The results of phase one experiment served as basis to select the suitable tree leaf extract for the reductive degradation of CT. The second phase of the study consisted of three sub-experiments. First was the investigation of the effect of tree leaf, iron, and initial pH on the solution's reductive potential and final pH. Second was the optimization process using Taguchi method wherein three factors (initial pH, iron dosage, and tree leaf dosage), each with three levels, were evaluated. The third sub-experiment was the identification of CT degradation by-products. The result was used to determine degradation pathway of CT. The last phase of the study was the application of the optimized condition to degrade CT in actual field groundwater. This was to assess the robustness of the design used and the effectiveness of the treatment method in an actual setting considering the alkalinity, hardness, presence of cations and anions in the groundwater.

#### 2.2.1 Preparation of tree leaf extract

All leaves were collected on the same day, thoroughly rinsed with DI water, and then dried in an oven at 50 °C for 24 h. Once dried, the leaves were cut into small pieces to ensure they could pass through a #8 mesh sieve. 25 g of tree leaves were soaked in 500 mL DI water, achieving a concentration of 50 g L^−1^ and then the mixture was stored in a chamber at 20 °C for 24 h to allow extraction. Thereafter, the mixture was sieved to remove the leaves and then vacuum filtered through a 0.45 μm mixed cellulose membrane filter (ADVANTEC). The filtrate extracts were stored in glass bottles at 20 °C in the dark prior to experimentation and analysis. The experimental design for analysis of properties of tree leaves is presented in Table S1 (ESI).†

#### 2.2.2 Preparation of CT solution with varying pH

Three pH conditions (7, 9, and 10) were investigated. The pH 7 solution consisted of unbuffered DI water. The pH 9 and 10 solutions were buffered with carbonates, using 0.345 M NaHCO_3_/0.050 M Na_2_CO_3_ for pH 9 and 0.166 M NaHCO_3_/0.234 M Na_2_CO_3_ for pH 10. A CT solution (100 mg L^−1^) was prepared by directly injecting pure CT liquid into a 1 L glass bottle containing the pre-adjusted solutions with varying pHs. To prevent CT volatilization, zero headspace was maintained. The bottle was then stirred for 24 h at 20 °C in a temperature-controlled chamber.

#### 2.2.3 Preparation of TLE–CT reaction solutions

The CT/TLE-filtrate reaction was carried out by mixing equal volumes of a 100 mg per L CT solution and TLE-filtrate, resulting in a final concentration of 50 mg per L CT and up to 50 g per L TLE. The mixture was then distributed into a series of 20 mL reaction vials, each pre-dosed with 1 mL of concentrated ferrous sulfate heptahydrate solution. After the addition of the CT solution, the iron concentrations were diluted to the designated levels of 1 mM, 10 mM, or 15 mM. A detailed summary of the experimental factors and levels is provided in Table S2 (ESI).† To prevent volatilization, zero headspace was maintained in all vials, which were then placed in a temperature-controlled chamber at 20 °C. Before analysis, the reaction vials were centrifuged at 500 rpm for 30 min.

#### 2.2.4 Experimental optimization design

The Taguchi method was employed for optimization, as it determines each factor's contribution, establishes relationships between variables and conditions, and achieves optimal performance with a limited number of experiments.^25^ There were three factors considered (pH, leaf dose, and iron dose) and each factor has three levels, hence employing L9(3^3^) orthogonal array, which is presented in Table S2 (ESI).† The removal of CT was evaluated using the signal-to-noise (S/N) ratio, specifically applying the “larger-is-better” formula as expressed in eqn (1).^26^1
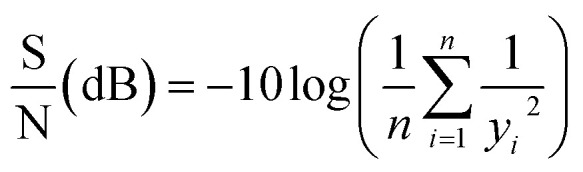
where *y*_*i*_ is result of the experiment and *n* is the number of experiments.

To determine which factor has significant influence on the response variable, the analysis of variance was used. The total variability of the S/N ratios was separated into contributions according to each parameter and error. The total sum of square (SS_T_), degrees of freedom (DOF), sum of squares (SS_A_), variance (*V*_e_), *F*-value, and contribution (%), were calculated using the eqn (2)–(7):^27,28^2
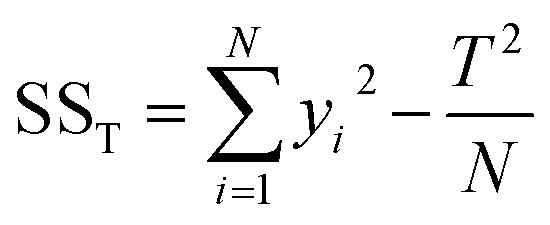

3 DOF = *N*_*i*_ − 1

4
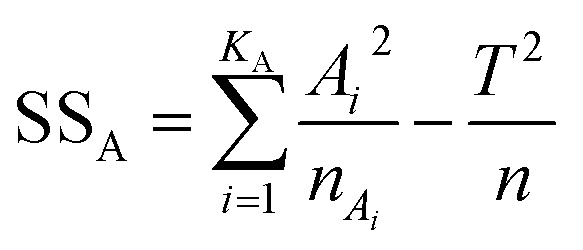
5
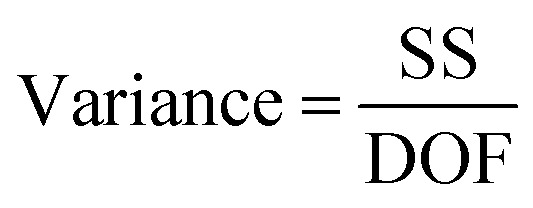
6
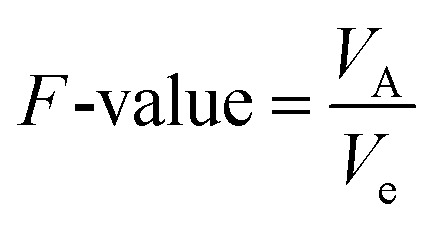
7
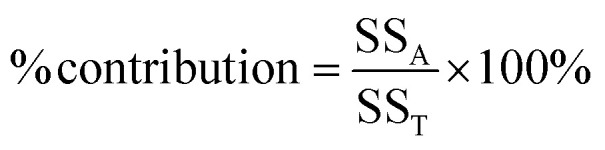
where *N*_*i*_ is the number of levels of the factor, SS_A_ is the sum of square of the factor *A*, *T* is the sum of S/N ratios, *K*_A_ is the level of factor *A*, *A*_*i*_ is the sum of S/N ratios of factor *A* at level *i*, *V*_A_ is the variance of the factor, and *V*_e_ is the variance of the error. To identify which factor is statistically significant, the *F*-test can be done by comparing the *F*-value of the parameter to the critical value at a given confidence interval.

After the selection of optimal parameters, the final step is the confirmation test, which involves prediction and verification of results using the optimal parameters. The S/N ratio (*η*) at the optimal process parameters can be determined using eqn (8):^27,28^8
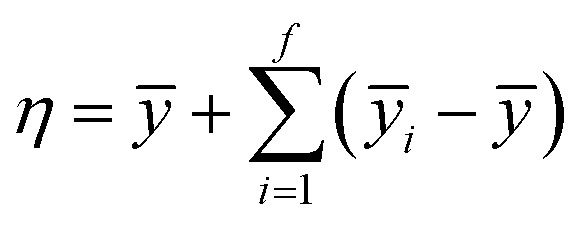
where *ȳ* represents the overall mean value of S/N ratios for all experimental results, *f* denotes the factor number, and *ȳ*_*i*_ is the mean of S/N ratio corresponding to targeted factor levels.

### 2.3 Analysis

The CT was analyzed using Gas Chromatography/Flame Ionization Detector (GC/FID) Agilent 7890B equipped with Purge and Trap (P&T, O.I. Analytical Eclipse 4660). After the aqueous sample was centrifuged, 5 mL of the supernatant was withdrawn, filtered using 0.45 μm PTFE syringe filter, and injected into the P&T to concentrate the volatile organic compounds. The column used was Agilent HP-5 (30 m × 0.32 mm, 0.25 μm). The analysis of CT degradation byproducts was carried out using gas chromatograph/mass spectrometer (Agilent 7890A GC/Agilent 5975C MS) with an Agilent J&W Scientific DB 624 column (60 m × 0.25 mm i.d.) in electron impact mode and equipped with P&T instrument (O.I. Eclipse 4760). Anions and cations were analyzed using ion chromatography instrument (Metrohm 790) with the column SH-AC-4 column (4.6 × 250 mm) and Metrosep C4-250/4.0, respectively. The analytical procedures and corresponding references for the characterization of tree leaf properties, including antioxidant capacity, reducing power, chelating activity, and total polyphenol content, as well as the qualitative analysis of polyphenol constituents, are summarized in Table S3 (ESI).† The ferrous ion and total iron concentrations were measured using a spectrophotometric method, as described in Table S3 (ESI).†

## 3. Results and discussion

### 3.1 Characterizations of tree leaves

**Fig. 1:**
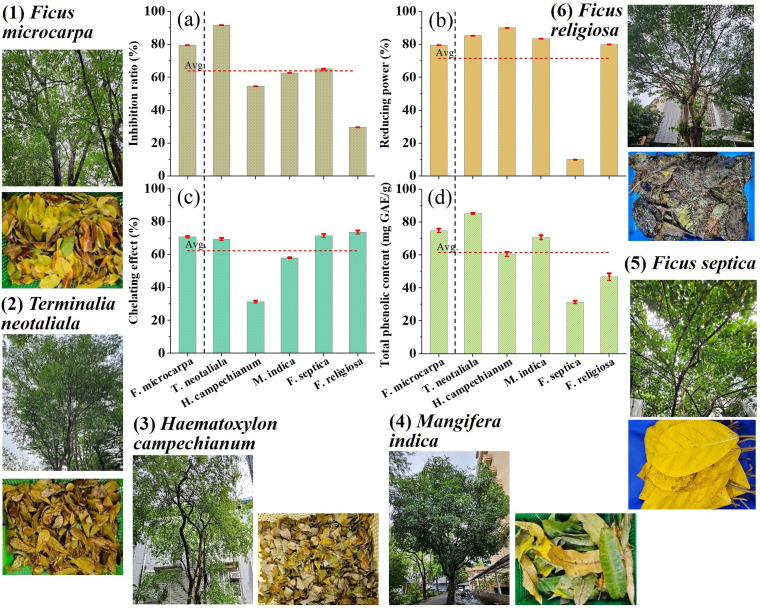
Characteristics of TLE (a) antioxidant capacity, (b) reducing power, (c) metal chelating capacity, and (d) total phenolic content. Inset shows pictures of the trees and their corresponding waste leaves for (1) FM, (2) TN, (3) HC, (4) MI, (5) FS, and (6) FR, respectively.

The basic properties of tree leaves, including antioxidant capacity, reducing power, chelating effect, and total phenolic content are shown in Fig. 1. The antioxidant assay used linoleic acid, which can be oxidized by oxygen species present in water. The phenolic compounds present in TLE can prevent the oxidation process. Among the six tested tree leaf extracts, *Terminalia neotaliala* has the highest antioxidant capacity (92% inhibition), followed by *Ficus microcarpa* (79%), as shown in Fig. 1(a). Fig. 1(b) presents the reducing power assay results, where *Haematoxylon campechianum* and *Terminalia neotaliala* extracts demonstrated the strongest reducing power, with values of 90% and 85%, respectively. Reducing power assay is based on the principle that substances with reduction potential reduce potassium ferricyanide into potassium ferrocyanide, which then reacts to form ferric–ferrous complex.^29^ A higher percentage of reducing power indicates a stronger reducing potential.

Fig. 1(c) shows the metal chelating ability expressed by the chelating effect. Among the six extracts, *Ficus religiosa* extract exhibits the highest chelating ability at approximately 74%, then *Ficus septica* with 71.5%. In the assay, iron tetrachloride was added to the plant extract sample followed by ferrozine. The phenolic compounds bind with certain amount of Fe^2+^, but the remaining Fe^2+^ reacts with ferrozine. In the presence of phenolic compounds, there is inhibition of ferrozine–Fe^2+^ complex formation due to binding of Fe^2+^ to phenolic structures, which leads to the decrease in absorbance value.^30^ The lower the absorbance value, the higher is the chelating ability. The last property is the TPC, which is expressed in mg GAE per g dry leaves. In this assay, Folin–Ciocalteu reagent was used to oxidize phenolic groups such as gallic acid to reduce heteropoly acid (phosphomolybdate–phosphotungstate) contained in Folin–Ciocalteu reagent into a molybdenum–tungsten complex, which is blue in color and can be measured at 765 nm. Sodium carbonate was added to the mixture because the combined phenolic can only be supported by Folin–Ciocalteu reagents in an alkaline environment so that protons dissociate in phenolic compounds into phenolic ions. As the concentration of phenolic ions increases, more heteropoly acid is reduced, resulting in a more intense blue color.^31^ Based on the result shown in Fig. 1(d), *Terminalia neotaliala* extract exhibits the highest TPC value of approximately 85 mg GAE per g dry leaves. The TPC values obtained from the six tree leaf extracts in this study ranged from 30 to 85 mg GAE per g (Fig. 1(d)), which fall within the reported range of 19–201 mg GAE per g for 18 indigenous tree species in Taiwan.^32^

The quantitative analysis of the four properties was used to determine the most suitable TLE for the reductive degradation of carbon tetrachloride. Based on the results, *Terminalia neotaliala* extract emerged as the optimal choice for CT degradation, as it consistently showed higher values in two of the screening criteria (*i.e.*, antioxidant capacity and reducing power) and performed above average across all four properties.

The solubility and extractability of polyphenols from tree leaves are largely influenced by their structural characteristics. Phenolic acids, which contain hydroxyl and carboxyl functional groups, are generally water-soluble. In contrast, flavonoids, lignans, and stilbenes exhibit low water solubility due to their multiple aromatic rings, non-polar bonds, and relatively high molecular weights. To extract a broader range of polyphenols, polar organic solvents such as acetone, ethanol, and methanol are commonly employed. Among these, ethanol is widely regarded as a safe and effective solvent and is often used in combination with water in various proportions.^33^ Traditional extraction techniques like solvent-liquid extraction and Soxhlet extraction are widely used but come with limitations, including high solvent consumption, energy demand, and long processing times. More sustainable and efficient alternatives, such as microwave-assisted extraction and supercritical CO_2_ extraction, offer advantages including reduced solvent use, better energy efficiency, improved temperature control (which helps preserve polyphenol stability), and higher purity yields.^34^ In the context of this study, the extracted polyphenols are intended for subsurface remediation and would potentially be injected into groundwater. Therefore, water-soluble polyphenols are preferred to ensure compatibility and effective distribution in the aqueous subsurface environment. Nevertheless, exploring different solvent extraction methods remains valuable for isolating a wider variety of polyphenol compounds for potential applications beyond water-based systems.

#### 3.1.1 Qualitative determination of polyphenols

**Fig. 2:**
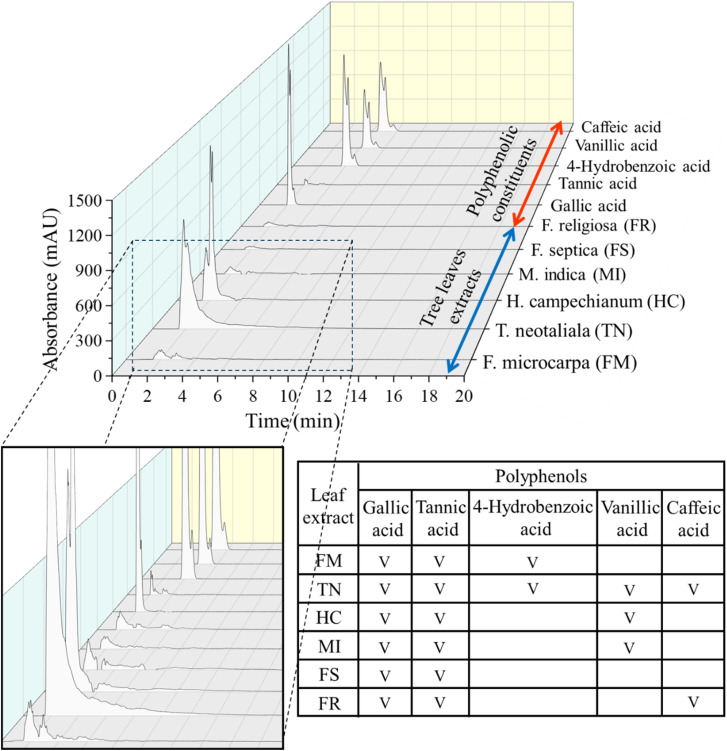
HPLC/PDA chromatogram at 254 nm of polyphenolic constituents and tree leaves extracts. The inserted table summarizes the identified polyphenols present in the corresponding tree leaves.

As shown in Fig. 2, gallic acid is the predominant compound in all the tree leaf extracts, with especially high signals observed in TN and HC. Gallic acid (3,4,5-trihydroxybenzoic acid), a naturally occurring low-molecular-weight triphenolic compound, is widely abundant in many plant species. Ahani and Khatibzadeh^35^ reported gallic acid as an effective reducing agent and capping agent that prevents other substance from rapid oxidation. According to Spiegel *et al.*,^36^ the presence of two or more –OH positioned *ortho* or *para* to each other leads to high antioxidant capacity. When the second –OH group is positioned in the *ortho* or *para* configuration, it becomes activated as the oxygen atom participates in the delocalization or distribution of electrons. Therefore, reduction process further proceeds. In gallic acid, 3 hydroxyl groups are in adjacent positions, which confirm that it can significantly inhibit the process of oxidation and able to scavenge free radicals. The other common polyphenol compound present in the TLE is the tannic acid. Tannic acid, a member of the tannin family, features a central glucose molecule esterified at all its hydroxyl groups with 10 gallic acid units. Its numerous hydroxyl groups contribute to its strong radical-scavenging capacity and potent antioxidant capacity.

The 4-hydrobenzoic acid, which is observed in TN and FM, has hydroxy substituent positioned at C-4. Carboxyl group does not benefit radical scavenging because of its electron-withdrawing nature. But upon the carboxyl deprotonated, it becomes electron donating group.^37^ Vanillic acid has a methoxy group at C-3. Based on the result, vanillic acid was detected in TN and HC. According to Spiegel *et al.*,^36^ methylated compounds tend to exhibit lower antioxidant capacity than their non-methylated counterparts due to low active electron- and hydrogen-donating groups. Lastly, caffeic acid appeared to be present in the TN and FR. Caffeic acid is a derivative of cinnamic acid, featuring hydroxy groups at the 3 and 4 positions of the phenyl ring. Caffeic acid is reported to exhibit superior antioxidant capacity compared to other hydroxycinnamic acids, a property attributed to its enhanced radical stability achieved through extended π-electron delocalization and hydrogen bonding with the vicinal hydroxyl group formed following hydrogen atom transfer.^38^

**Fig. 3:**
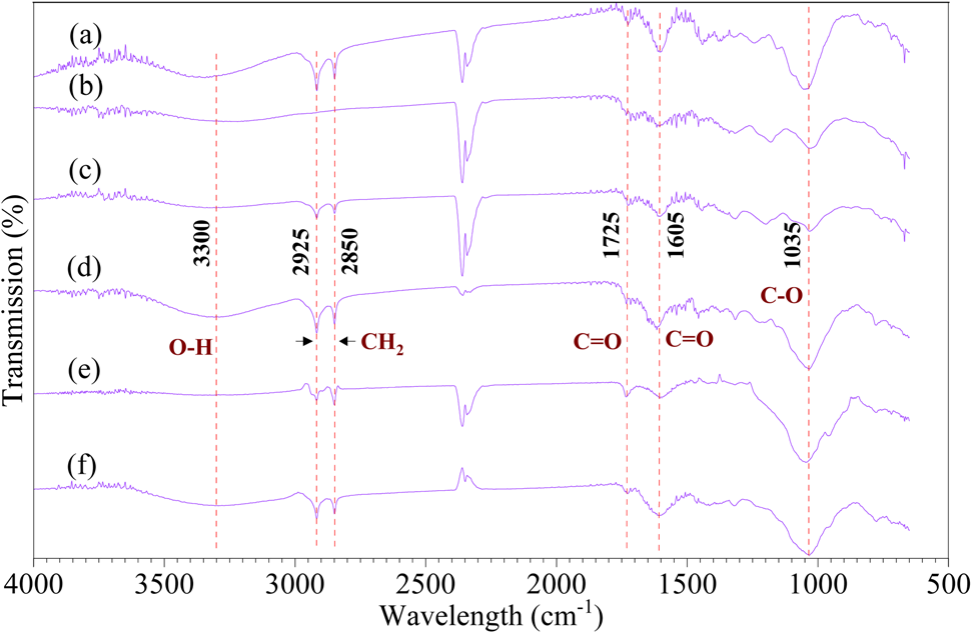
FTIR-ATR spectra of (a) FM, (b) TN, (c) HC, (d) MI, (e) FS, and (f) FR.

Fig. 3 shows the FTIR spectra of the six tree leaves. The broad peak at 3300 cm^−1^ is related to the –OH group, attributed to the presence of hydroxyl groups in the molecules of tree leaf extract polyphenol.^10,39^ The peaks at 2925 and 2850 cm^−1^ correspond to vibrations of methyl (CH_2_),^39,40^ likely from substances such as vanillic acid. A peak at 1725 cm^−1^ is due to the bending vibration of carbonyl (C=O) in the carboxyl group (–COOH), associated with compounds like 4-hydroxybenzoic acid. Additionally, a peak at 1605 cm^−1^, is related to the stretching of the carbonyl group such as in flavanols.^39,41^ Because flavanols are only slightly soluble in water, they may go undetected in plant extracts prepared exclusively with water. The peak at 1035 cm^−1^ is associated with C–O bonds in alcohol groups.^39^ Leaf extracts that exhibited higher Fe^2+^ chelation capacity, such as *Terminalia neotaliala*, showed stronger absorption bands corresponding to hydroxyl (–OH) and carbonyl (C=O) functional groups. These groups are known to play key roles in metal chelation due to their electron-donating ability. Specifically, phenolic –OH groups can coordinate with Fe^2+^ by donating lone pair electrons from the oxygen atom to the vacant d-orbitals of iron, forming stable chelate rings. Likewise, carbonyl groups, particularly in conjugated systems such as flavonoids, can act as bidentate ligands by coordinating through both carbonyl oxygen and adjacent hydroxyl groups, enhancing complex stability. This chelation mechanism has been documented in studies on polyphenol–metal interactions.^42,43^ The presence of these functional groups in higher density and accessibility likely contributes to the superior chelation activity observed in *Terminalia neotaliala*. This interpretation helps explain the differences in chelation efficiency across samples and is consistent with previous findings that polyphenolic structures with *ortho*-dihydroxy or carbonyl–hydroxyl motifs exhibit stronger metal binding affinity.

#### 3.1.2 Effect of tree leaf, iron, and initial pH to the ORP and pH of solution

**Fig. 4:**
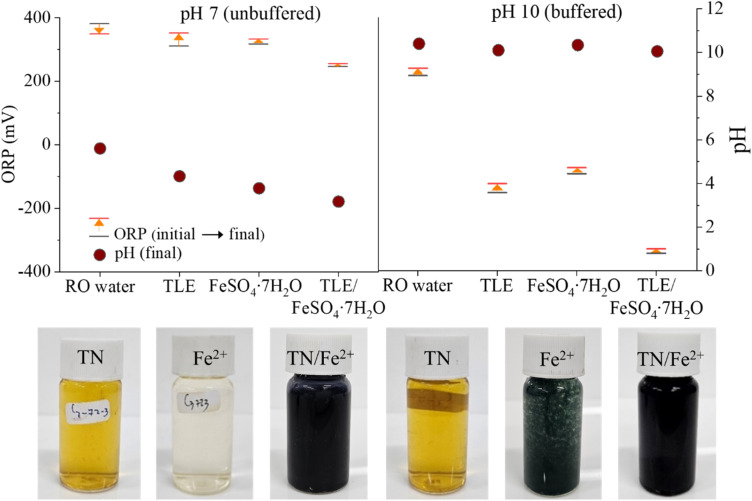
pH and ORP profile of tree leaf extract, Fe^2+^ additive, and TLE–Fe at pH 7 (unbuffered) and pH 10 (carbonate buffered). Insets show the picture of reaction solutions.

Phenolic acids exhibit weak antioxidant capacity under acidic conditions, but this property increases with increasing pH due to the ionization of hydroxyl group.^44^ Deprotonation in hydroxyl group, which induced electron-donating ability of polyphenol compounds occurs when the pH is greater than the dissociation constant p*K*_a_ value. Based on the p*K*_a1_ and p*K*_a2_ values of most phenolic acids, which fall between 4 and 10,^45^ this experiment evaluated two pH conditions, pH 7 and 10, using a TN extract solution noted for its high abundance of polyphenolic constituents as the model system. The ORP can indicate whether an aqueous solution possesses antioxidant properties. A negative ORP typically indicates a highly reducing environment, implying a greater capacity for the reductive degradation of CT. As shown in the Fig. 4, test conditions at pH 7 displayed positive ORP values, even when iron and TN extract were combined. A decrease in pH, such as in NT extract where the pH dropped from 7 to 4.5, suggests deprotonation occurs to release H^+^, resulting in lowering pH. As seen in Fig. 4, at pH 10, polyphenol in the TLE at this higher pH exhibits a negative ORP of −167 mV and the iron additive transformed into green rust, which acts as a strong reducing agent with an ORP value of −116 mV. The leaf extract is yellow under neutral and alkaline conditions. When combined with Fe^2+^, their ORP decreases further to −379 mV, more than doubling their individual reductive potentials. The black color observed in the combination of iron and TN extract indicates the formation of a polyphenol–iron complex. These findings indicate that the polyphenol and iron complex promote a more reducing environment, which exhibits the potential in reductive degradation of CT. The formation of polyphenol–iron complexes was visually evident in this study by the appearance of a dark black solution, as shown in Fig. 4. This observation is consistent with previous reports describing similar complexation between iron and plant-derived polyphenols. For instance, Markova, *et al.*^46^ reported the formation of green tea polyphenol–Fe complexes exhibiting comparable visual characteristics. Similarly, Wang *et al.*^47^ observed analogous complexation behavior between iron and eucalyptus leaf extracts. While UV-Vis spectroscopy was performed to further investigate the formation of these complexes, the results did not conclusively confirm their presence, possibly due to spectral overlaps or matrix effects. Direct structural identification using advanced techniques such as X-ray absorption spectroscopy (XAS) or Electron Spin Resonance (ESR) was not feasible in the current study but is recommended for future work to substantiate the formation and structure of these complexes. Nevertheless, the consistent visual evidence with prior literature supports the likely occurrence of polyphenol–iron interactions under the conditions examined.

### 3.2 Optimization of leaf dose, pH, and iron dose using Taguchi orthogonal array for the reductive degradation of CT

**Fig. 5:**
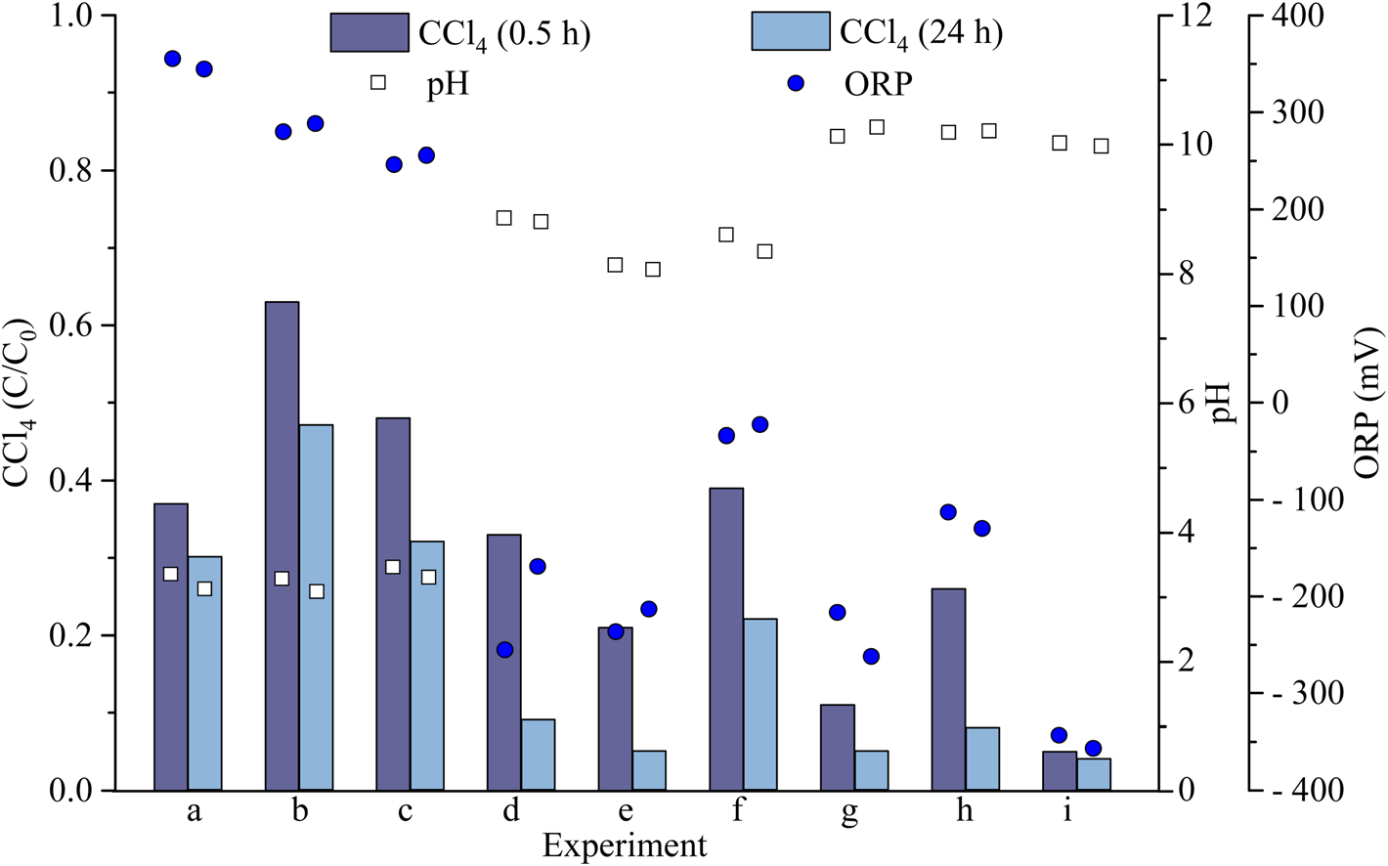
Concentration variations of CT and changes of pH and ORP after degradations at designated reaction conditions [CT]_0_ = 50 mg L^−1^.

The concentration of CT in all experimental runs decreased after 24 h of reaction as shown in Fig. 5. The pH of experiments (a), (b), and (c) dropped to ∼3 from initial neutral pH after 30 min and slightly decreased further after 24 h. Experimental groups with the decrease of pH, their CT concentrations after reaction are higher compared to those experiments prepared in buffered alkaline solution. At initial neutral pH condition, increasing the leaf and iron doses resulted in the least removal of CT. There is only a slight difference in final CT concentration when both parameters are at their maximum or minimum levels. In addition, despite the non-reducing environment indicated by high positive ORP values, the removal of CT at an initial neutral pH ranged from about 53% to 70%.

When the pH exceeds the p*K*_a_ of polyphenols, such as gallic acid with p*K*_a1_ value of 4.24,^48^ the OH functional group dissociates, producing phenoxy radicals that release electrons to stabilize the structure. In addition, the oxidation of Fe^2+^ to Fe^3+^ releases electrons that cleave the C–Cl bond in CT, resulting in its degradation. At around neutral pH, the polyphenols present in TLE form complexes with Fe^3+^ particularly a biscomplex,^49^ in which two ligands (catecholate or gallate groups) of the polyphenols bind with iron. When a catecholate or gallate ligand binds to Fe^3+^, the polyphenol can reduce the iron to Fe^2+^, enabling its recycling within the system.^13^

Experiments (d), (e), and (f) were prepared at an initial buffered pH 9. At experiments (d) and (e), increasing leaf dose and iron dose were observed to increase removal of CT between 91% and 94%. When the iron dose was reduced while the leaf dose was increased as observed in experiment (f), the removal efficiency decreased to about 78%, suggesting that insufficient iron hindered the formation of stable polyphenol complexes. Negative ORP between −22 mV to −213 mV were also observed. On the other hand, experiments (g), (h), and (i) demonstrated relatively low final CT concentrations, achieving removal efficiencies between 92% and 96%. The low ORP values, ranging from −129 mV to −356 mV, indicated a highly reducing environment.

At alkaline condition, polyphenol and iron forms triscomplex, which has three ligand molecules in its coordination system. Triscomplex is stable and possess antioxidant capacity.^12,49^ Based on the results, CT removal was more effective under buffered alkaline conditions compared to experiments with an unbuffered neutral condition. Among all 9 experiments, experiments (g) and (i) achieved the highest removal efficiency at 95% and 96%, respectively, after 24 h. The Taguchi experimental design outcomes were analyzed to assess the contribution of each factor. The average CT removal was transformed into a signal-to-noise ratio using eqn (1), which served as the assessment index for evaluating the results.

**Fig. 6:**
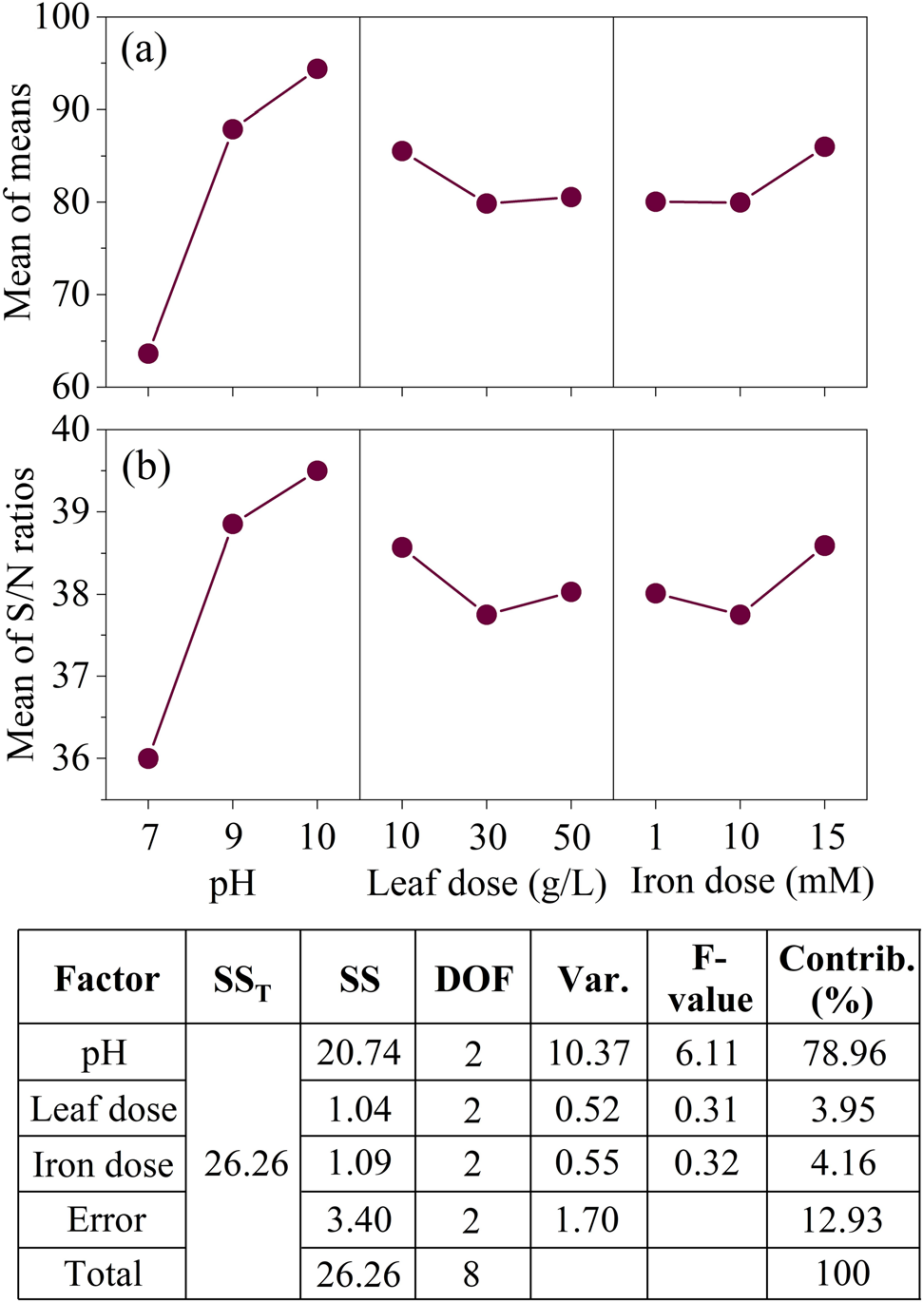
Plots showing the effects of each factor on the CT removal rate in the aqueous phase. (a) Average removal efficiencies of CT and (b) S/N ratios of process parameters. Inserted table shows analysis of variance for CT degradation.

As shown in Fig. 6 (data detailed in Table S4 (ESI)†), the optimal levels for the experimental factors in the reductive degradation of CT were identified as follows: pH at level 3 (S/N = 39.50), leaf dose at level 1 (S/N = 38.03), and iron dose at level 3 (S/N = 38.59). The trend suggests that increasing the pH and iron dose, while reducing the leaf dose, enhance CT degradation. Analysis of variance was employed to calculate the degrees of freedom, sum of squares, and variance for each factor, enabling the assessment of the relative importance of various parameters, including the *F*-value (degree of influence) and contribution percentage (statistical results tabulated in Fig. 6).

The calculated *F*-value can be compared to the critical value extracted from *F*-distribution table of 4.46 at 95% confidence interval (*α* = 0.05). The pH which *F*-value is 6.11 is found to be significant. The result indicates statistically significant difference in CT removal among pH values tested. Also based on the % contribution, pH has the highest influence on the CT removal accounting for 79%, followed by iron dose and then leaf dose. Eqn (8) is used to estimate the expected S/N ratio for a combination of factor levels identified as optimal based on the experimental data. By calculating the deviation of each optimal level's mean from the overall mean and summing them, this equation predicts the system's performance under optimal conditions. Accordingly, eqn (8) was applied to estimate the expected outcome of CT degradation under the identified optimal conditions. The corresponding predicted removal rate based on the calculated S/N ratio, is approximately 100%. This signifies that the Taguchi experimental design is effective in optimizing the parameters to achieve the best result.

#### 3.2.1 By-product formation and degradation pathway of carbon tetrachloride

**Fig. 7:**
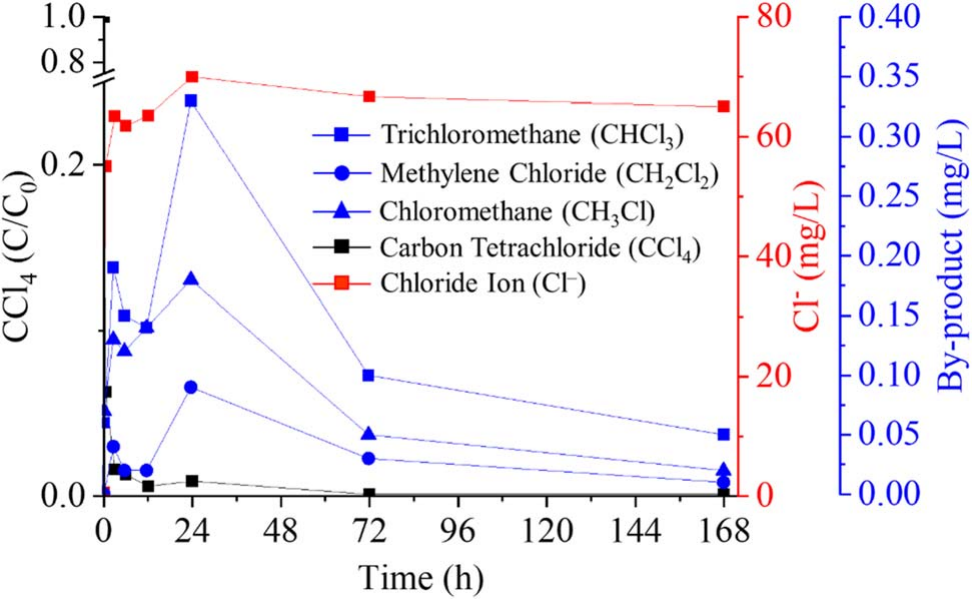
Concentration variations of CT and its degradation byproducts at optimal experimental conditions.

A confirmation test under the optimal experimental conditions, determined through statistical analysis of the Taguchi design results, was conducted to further investigate CT degradation by-products and the associated pathway. Fig. 7 illustrates the concentrations of CT, chloride ions, and generated by-products with reaction times. Three byproducts were detected using GC-MS, including trichloromethane (CHCl_3_), methylene chloride (CH_2_Cl_2_), and chloromethane (CH_3_Cl). The CT concentration exhibits a rapid decline, reaching approximately 6% of its initial value within 30 min. Further slight decrease in CT concentration is observed between 12 h and 24 h. After 168 h, nearly complete degradation is achieved, with a removal efficiency of 99.94%. In terms of by-product formation, trichloromethane was the primary by-product, reaching a peak concentration of 0.33 mg L^−1^ at 24 h, but decreased to approximately 0.05 mg L^−1^ after 168 h. This final concentration is below the Maximum Contaminant Level (MCL) of 0.08 mg L^−1^ for chloroform in drinking water set by the United States Environmental Protection Agency (USEPA). Chloromethane showed a maximum concentration of 0.18 mg L^−1^ at 24 h, which dropped to 0.02 mg L^−1^ after 168 h, which concentration falls below MCL in drinking water. Methylene chloride had a high concentration of 0.09 mg L^−1^ at 24 h, which then decreased to 0.01 mg L^−1^ after 168 h, a level exceeding the MCL of 0.005 mg L^−1^. Lastly, the chloride ion concentration was nearly zero initially but increased to 38.57 mg L^−1^ after 30 min and reached 45.53 mg L^−1^ after 168 h, indicating the liberation of chloride ions and complete mineralization of CT. This observation aligns with theoretical calculations showing that 50 mg L^−1^ (0.321 mM) of CT would release 46.09 mg L^−1^ of chloride upon complete mineralization, corresponding to a CT/Cl molar ratio of 4 : 1.

**Fig. 8:**
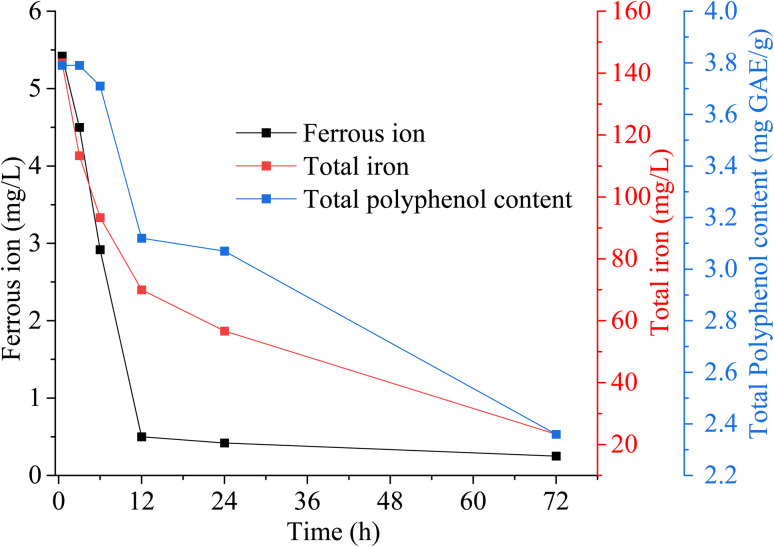
Concentration variations of polyphenol and iron at optimal experimental conditions.

Fig. 8 shows the concentration profiles of Fe^2+^, total iron, and polyphenols during the reaction. Initially, Fe^2+^ was about 8000 mg L^−1^, but it dropped rapidly to 5.42 mg L^−1^ within 30 min and was nearly depleted by 24 h. This rapid depletion hindered the formation of polyphenol–iron complexes, thereby slowing CT degradation, as evidenced by the spikes in CT and its by-products around 24 h (Fig. 7). Total iron was measured to track Fe^3+^ levels, given that Fe^3+^ concentration can be inferred from the difference between total iron and Fe^2+^. Notably, total iron (predominantly Fe^3+^) continued to decrease after 24 h, along with a decline in polyphenol content. At this stage, polyphenols and their Fe^3+^ complexes became the key drivers of CT degradation. The polyphenol–Fe^3+^ complex can regenerate Fe^2+^, thereby supplying the electrons necessary for continued CT degradation.

When the pH exceeds the p*K*_a_ of the polyphenols, the OH functional group dissociates, releasing H^+^ ions and subsequently emitting electrons (to stabilize the compound), which is transferred to CT. In addition, polyphenol forms coordination complex with Fe^2+^ but due to strong stabilization of Fe^3+^ by polyphenol ligands over Fe^2+^, the Fe^2+^–polyphenol complex undergoes rapid oxidation to produce Fe^3+^–polyphenol complex (triscomplex), releasing electrons in the process. The polyphenol in Fe^3+^–polyphenol complexes reduced Fe^3+^, therefore regenerating Fe^2+^. This Fe^2+^/Fe^3+^ cycle, which involves electron release, results in the degradation of CT.

**Fig. 9:**
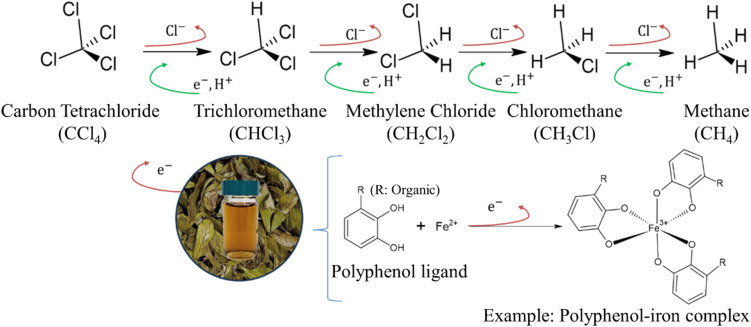
Proposed degradation pathway of CT with polyphenol–iron complex.

The reductive degradation of carbon tetrachloride (CT) by electrons released from ascorbic acid^5^ or guava leaf extract,^50^ either alone or in combination with dissolved Fe^2+^ or iron minerals to enhance electron transfer, proceeds *via* hydrogenolysis and dichloroelimination pathways. In hydrogenolysis, the cleavage of a C–Cl bond in CT occurs through a single-electron transfer, resulting in the formation of trichloromethane (CHCl_3_). Alternatively, dichloroelimination may occur through a two-electron transfer, producing radical intermediates rather than CHCl_3_. Both pathways ultimately lead to the reductive dechlorination of CT, releasing four chloride ions. The rapid and substantial formation of CHCl_3_, followed by the appearance of CH_2_Cl_2_ and CH_3_Cl in the reaction system, suggests that hydrogenolysis may be the dominant degradation pathway. Accordingly, the degradation of CT using tea leaf extract in the presence of iron likely involves both hydrogenolysis and dichloroelimination mechanisms. The proposed degradation process involving polyphenol–iron complexes is illustrated in Fig. 9. In the hydrogenolysis pathway, CT accepts electrons, leading to C–Cl bond cleavage and substitution of chlorine with hydrogen to form CHCl_3_. The subsequent detection of intermediates such as CHCl_2_, CH_2_Cl_2_, and CH_3_Cl further supports the occurrence of sequential electron transfer steps. It is speculated that the final degradation product is methane (CH_4_), accompanied by the release of chloride ions. However, because qualitative analysis of intermediate compounds was not performed in this study, distinguishing between the hydrogenolysis and dichloroelimination pathways remains uncertain and warrants further investigation. During the reductive degradation of CT, a cascade of sequential hydrogenolysis reactions may occur, often resulting in the accumulation of partially dechlorinated intermediates such as chloroform, methylene chloride, and methyl chloride. These intermediates pose significant environmental and health risks. For example, methylene chloride is classified as a potential human carcinogen and has relatively high mobility in subsurface environments, increasing the likelihood of groundwater contamination.^51^ In some cases, concentrations of these intermediates may exceed regulatory limits, undermining the effectiveness of *in situ* remediation strategies. To mitigate the formation and persistence of these toxic by-products, one essential approach is to ensure complete reductive dechlorination. This involves optimizing polyphenol–iron complex electron donor availability, maintaining an appropriate redox environment, and ensuring sustained reaction conditions that promote full transformation of CT to non-toxic end-products such as methane and chloride ions. Additionally, a sequential treatment strategy could be employed. For example, an initial abiotic phase driven by polyphenol–iron complexes can be followed by a biotic phase utilizing specialized anaerobic microbial consortia capable of cometabolizing or reductively dechlorinating chloroform and methylene chloride.^52,53^ Overall, designing integrated treatment systems that combine abiotic and biotic mechanisms offers a more robust and sustainable strategy for minimizing the accumulation of toxic intermediates and achieving regulatory compliance in field-scale applications.

#### 3.2.2 Carbon tetrachloride degradation in groundwater solution

The optimal conditions were applied to the groundwater sample that has properties detailed in Table 1. The groundwater has a neutral pH, and its ORP is approximately three times lower than that of DI water. The total dissolved iron is present in trace concentrations. All other parameters fall within the typical or acceptable range for groundwater. In the controlled system where RI water was used for the degradation experiment, these parameters were eliminated or minimized, allowing for the analysis of variables without confounding interactions. However, to evaluate the effectiveness of the treatment method, it is important to apply it in a real-world system.

**Table 1:** Properties of groundwater

Property	Value
pH	6.79
ORP (mV)	101.63
DO (mg L^−1^)	4.01
Total dissolved iron (mg L^−1^)	0.28
Fe^2+^ (mg L^−1^)	0.19
Fe^3+^ (mg L^−1^)	0.09
Na^+^ (mg L^−1^)	14.77
K^+^ (mg L^−1^)	2.31
Ca^+^ (mg L^−1^)	70.38
Mg^+^ (mg L^−1^)	14.00
Cl^−^ (mg L^−1^)	14.33
F^−^ (mg L^−1^)	0.39
SO_4_^2−^ (mg L^−1^)	103.31
Br^−^ (mg L^−1^)	Not detected
PO_4_^3−^ (mg L^−1^)	Not detected
NO_3_^−^ (mg L^−1^)	0.14
NO_2_^−^ (mg L^−1^)	Not detected
Alkalinity (mg CaCO_3_ L^−1^)	135
Hardness (mg CaCO_3_ L^−1^)	234

**Fig. 10:**
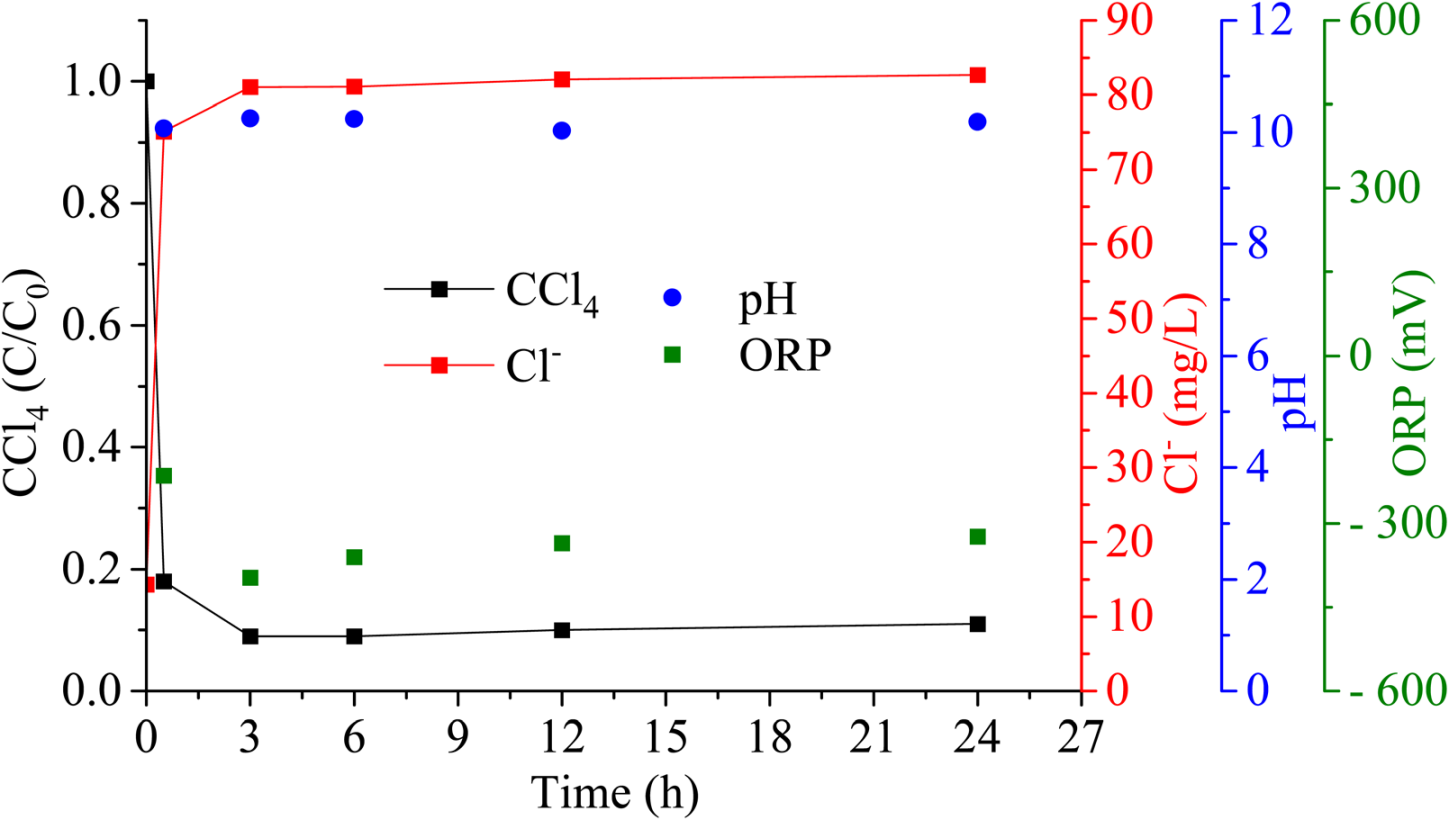
CT degradation in groundwater. Initial CT concentration is 50 mg L^−1^.

Fig. 10 illustrates the CT removal, chloride ion generation, and the profiles of ORP and pH over various reaction times. The concentration of CT exhibited a rapid decrease within the first 30 min, consistent with previous observations. The removal efficiency of CT in groundwater after 24 h measured 89%, which is lower than the RO water solution experiment achieving 95% of CT removal. This effect may be attributed to the ionic composition of the groundwater (Table 1), which can influence the formation and reactivity of polyphenol–iron complexes critical for the reductive degradation of CT. The groundwater contained both Fe^2+^ and Fe^3+^, as well as Na^+^, K^+^, Ca^2+^, and Mg^2+^. While iron plays a beneficial role by forming reactive complexes with polyphenols, the non-redox-active cations (Na^+^, K^+^, Ca^2+^, Mg^2+^) can alter the solution's ionic strength, potentially affecting the interaction between reductants and contaminants. The identified anions, Cl^−^, F^−^, SO_4_^2−^, and NO_3_^−^, may also impact the system. Both sulfate and nitrate are electron acceptors and may compete with CT for available electrons. Additionally, the groundwater's alkalinity implies the presence of carbonate and bicarbonate ions, which can precipitate iron and thereby potentially reduce the effectiveness of reductive processes. Chloride level rose to 57.86 mg L^−1^, in which background groundwater contains 14.33 mg L^−1^. The findings indicate that tree leaf polyphenols, when combined with iron, effectively degrade CT, highlighting their potential for practical applications. A preliminary test using actual groundwater to assess the reactivity of polyphenol–Fe complexes in degrading CT demonstrated successful reductive degradation, indicating that common groundwater constituents such as bicarbonate and sulfate did not inhibit complex formation or reactivity under these conditions. However, certain anions capable of competing for iron binding, such as phosphate, carbonate, and sulfate, may influence complexation dynamics to varying extents. A detailed investigation of these effects is recommended for future studies to optimize field applications.

While a pH of 10 condition enhances the reactivity of the polyphenol–iron complex, it presents several challenges for field remediation application. Maintaining elevated pH levels *in situ* requires frequent injection of soluble buffers, such as bicarbonates or carbonates, which may be rapidly consumed or transported beyond the target treatment zone.^54^ High pH conditions can also promote the precipitation of metals and minerals, potentially clogging aquifer pores, reducing permeability, and impairing reagent transport, which may result in uneven treatment distribution.^55^ Furthermore, elevated pH can inhibit native microbial communities, many of which thrive in the pH range of 6.5–8.5 and play a key role in natural attenuation of groundwater contaminants. Therefore, field applications must carefully consider the choice and dosage of buffering agents for pH control, the mobility and delivery method of reagents in the subsurface, and the compatibility of treatment conditions with indigenous microbial populations. In addition to commercial buffers, naturally occurring silicate minerals and carbonate rocks may offer long-term pH buffering. To improve reagent delivery, approaches such as recirculation wells or multi-point injection designs may be employed. Although polyphenol–iron complexes have shown no ecotoxicological effects,^46^ site-specific microbial assessments are essential to ensure treatment compatibility with the subsurface ecosystem.

The stability of polyphenols in extracts is a critical factor influencing the reproducibility and scalability of polyphenol-based remediation technologies. Their stability depends on both molecular structure and environmental conditions such as pH and temperature. Polyphenols with a greater number of hydroxyl groups are generally less stable due to their increased susceptibility to oxidation. For instance, compounds with a pyrogallol structure (*e.g.*, gallic acid, tannic acid, epigallocatechin gallate) are more readily oxidized than those with a catechol structure (*e.g.*, catechin, caffeic acid, epicatechin).^56^ Regarding pH, polyphenols tend to be more stable in acidic environments, whereas in alkaline conditions, they are prone to degradation, dimerization, and oxidation.^57^ Temperature also plays a crucial role, with lower storage temperatures helping to preserve polyphenol integrity. Salazar-Orbea *et al.*^58^ reported that storing polyphenols at temperatures between −20 °C and 4 °C can significantly slow their degradation, allowing preservation for up to 12 months. Given that different polyphenols respond variably to environmental factors, a comprehensive understanding of these influences is essential for assessing and optimizing stability.

## 4. Conclusion

The extract of waste tree leaves, in combination with ferrous ions, was utilized for the degradation of CT in an aqueous solution. The polyphenolic compounds present in *Ficus microcarpa*, *Terminalia neotaliala*, *Haematoxylon campechianum*, *Mangifera indica*, *Ficus septica*, and *Ficus religiosa* were analyzed to assess their reductive activities. *Terminalia neotaliala* was identified as the most effective candidate for CT degradation due to its high antioxidant capacity, reducing power, total phenolic content, and metal-chelating ability. The leaf extract was found to contain a range of compounds, including gallic acid, 4-hydroxybenzoic acid, vanillic acid, caffeic acid, and tannic acid.

The effect of leaf dose, iron dose, and pH in the degradation of CT in an aqueous solution were examined and optimized using Taguchi method employing an L9(3^3^) orthogonal array. The optimal conditions identified were pH 10, a leaf dose of 10 g L^−1^, and an iron dose of 15 mM. Analysis of variance determined pH was identified to be the most significant factor influencing the CT removal. CT undergoes sequential reductive degradation by accepting electrons and gradually releasing chlorine ions. After 24 h, the removal efficiency of CT reached 99% in an aqueous solution and 89% in field groundwater. Intermediate byproducts detected *via* GC/MS included CHCl_3_, CH_2_Cl_2_, and CH_3_Cl. This study concluded that waste tree leaves from *Terminalia neotaliala* serve as a potential source of polyphenols capable of reacting with iron to form coordination complexes, facilitating the reductive degradation of CT.

Although pH 10 was found to be optimal, investigating CT removal under unbuffered neutral conditions using tree leaf polyphenol–iron is recommended, as it may offer a more feasible and natural approach. Additionally, factors such as extended reaction time and temperature can be explored to enhance the reaction efficiency under neutral pH condition. Additional research is required to evaluate how common groundwater ions affect CT removal. These ions may reduce electron availability and participate in coordination complex formation, potentially explaining the lower CT removal efficiency in groundwater compared to pure water. Moreover, alternative methods for extracting polyphenolic compounds from tree leaves should be developed, followed by qualitative and quantitative analyses of their composition. Certain polyphenols, such as flavonoids, have limited water solubility, meaning they may not have been fully captured using the extraction method employed in this study. Lastly, applying tree leaf extracts for the degradation of other pollutants is recommended, as this approach could contribute to agricultural waste recycling and offer innovative solutions for pollution remediation.

Future research should explore the broader applicability of the polyphenol–iron complex system to other classes of groundwater contaminants, particularly those containing carbon or nitrogen in positive oxidation states that are amenable to reductive transformation, such as chlorinated ethanes, ethenes, and nitroaromatic compounds. Investigations under a range of geochemical conditions, including varying pH, redox potential, and natural organic matter content, will be critical for assessing the system's robustness and adaptability. Given that this reductive system is dependent on the formation of polyphenol–iron complexes, pH buffering plays a key role in facilitating electron release. Pretreatment strategies to adjust subsurface pH, such as the application of alkaline soil conditioners like limestone, may be beneficial in enhancing alkaline buffering capacity. In cases where pH buffering is insufficient, the intrinsic reducing capability of polyphenols or their complexes may serve as electron donors, creating a favorable redox environment to support subsequent biotic or abiotic remediation processes. Finally, field-scale validation and long-term performance evaluations are essential to advance the practical implementation of this sustainable remediation approach.

## Conflicts of interest

There are no conflicts to declare.

## Supplementary Material

RA-015-D5RA01391G-s001

## Data Availability

The data that support the findings of this study are available on request from the corresponding author, Chenju Liang.
