# Supplementary material for: Reductive degradation of carbon tetrachloride using tree leaf polyphenol–iron complexes for groundwater remediation
Source: RSC Adv. 2025 Jul 4;15(28):22915–29. doi: 10.1039/d5ra01391g (PMC12231589; doi:10.1039/d5ra01391g)
Supplement: RA-015-D5RA01391G-s001 [file RA-015-D5RA01391G-s001.pdf]

Supplementary Information (SI) for RSC Advances.
This journal is © The Royal Society of Chemistry 2025

Supplementary Materials

Reductive Degradation of Carbon Tetrachloride Using Tree Leaf Polyphenol-Iron Complex for Groundwater Remediation

Roselle Colastre Lasagas[1,2], Chenju Liang[1,*], Xuyen Thi Hong Luong[1], Florencio Ballesteros Jr.[3]

[1]Department of Environmental Engineering, National Chung Hsing University, 145 Xingda Road, South Dist., Taichung City 402202, Taiwan

[2]Environmental Engineering Graduate Program, University of the Philippines Diliman

[3]Department of Chemical Engineering, University of the Philippines Diliman

*Corresponding author. Tel.: +886-4-22856610; Fax: +886-4-22856610

E-mail address: cliang@nchu.edu.tw

Contents

Figure S1. Experimental flow chart
Table S1. Experimental design for analysis of basic properties of leaves.
Table S2. L9 Orthogonal array of CT degradation using polyphenol-iron complex.
Table S3. Qualitative analysis of antioxidation capacity, reducing power, chelating effect, and total polyphenol content, and qualitative analysis of tree leaf polyphenol constituents.
Table S4. The response value of each factor in the degradation of CT within the aqueous phase.

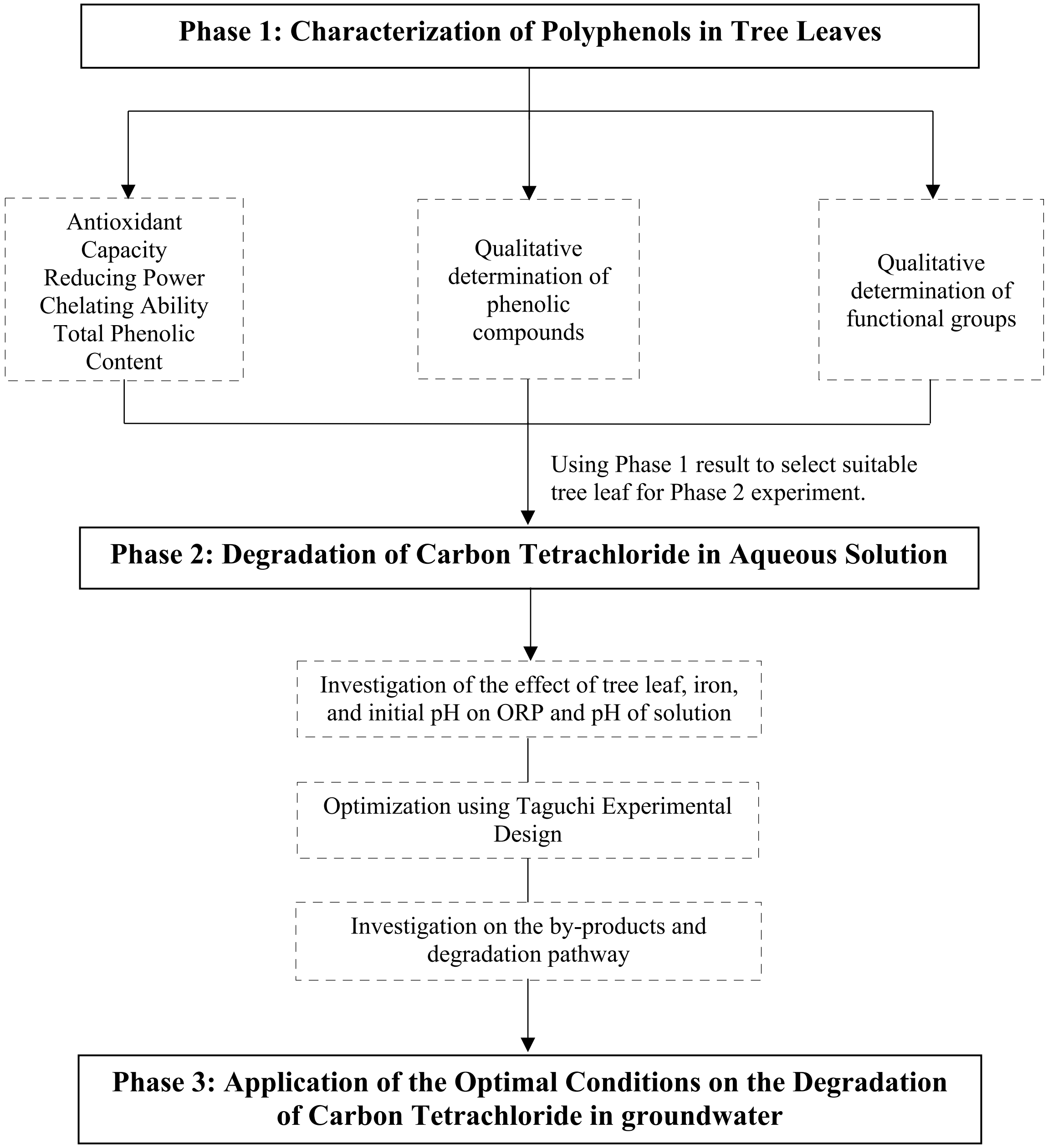

Figure S1. Experimental flow chart.

Table S1. Experimental design for analysis of basic properties of leaves.

Family	Leaves	Leaf solution	Parameters
Moraceae	*Ficus microcarpa*	50 g/L at 20 °C for 24 hours	Inhibition ratio<br>Reducing power<br>Chelating ability<br>Total phenolic content
Combretaceae	*Terminalia neotaliala*		
Fabaceae	*Hematoxylon camphecianum*		
Anacardiaceae	*Mangifera indica*		
Moraceae	*Ficus septica*		
Moraceae	*Ficus religiosa*		

Table S2. L9 Orthogonal array of CT degradation using polyphenol-iron complex.

	Factor		
	pH	Leaf dose (g/L)	$FeSO_4 \cdot 7H_2O$ (mM)
Level 1	7	10	1
Level 2	9	30	10
Level 3	10	50	15
Experiment			
a	7	10	1
b	7	30	10
c	7	50	15
d	9	10	10
e	9	30	15
f	9	50	1
g	10	10	15
h	10	30	1
i	10	50	10

Table S3. Qualitative analysis of antioxidation capacity, reducing power, chelating effect, and total polyphenol content, and qualitative analysis of tree leaf polyphenol constituents.

Properties	Analytical Procedures
Antioxidation capacity	The antioxidation capacity was conducted in accordance with the procedure of Yen & Chen (Yen and Chen, 1995). 0.1 mL of leaf extract (50 g of dry leaves L^{-1}) was added with 2.0 mL of linoleic acid emulsion (0.01 M, pH 6.6 phosphate buffer) and incubated in darkness at 37 °C to allow oxidation for 15 h. Before and after the oxidation process, 0.2 mL of mixture was mixed with 8.0 mL of 80% methanol and the absorbance of the resulting solution was measured at 234 nm using ultraviolet–visible spectrometer (PERSEE T6U model) and compared to the absorbance of ascorbic acid. The antioxidant capacity is expressed as inhibition ratio. $$\text{Inhibition ratio }(\%) = \left[1 - \frac{\Delta A_{234\ nm}\text{ sample (0 h - 15 h)}}{\Delta A_{234\ nm}\text{ control (0 h - 15 h)}}\right] \times 100$$
Reducing power	The reducing power was conducted according to the method of Lin et al. (2008). 2.5 mL of TLE solution (50 g of dry leaves L^{-1}) was added with 2.5 mL of phosphate buffer solution (0.2 M at pH 6.6) and 2.5 mL of 1% potassium ferricyanide. The resulting solution was placed in water bath at 50 °C for 20 min. Then, 2.5 mL of 10% of trichloroacetic acid solution was added to the mixture. 5 mL of the supernatant was mixed with 5 mL of DI water and 1 mL of 0.1% iron (III) chloride hexahydrate solution, and the absorbance was measured at 600 nm. Ascorbic acid was used as control. $$\text{Reducing power }(\%) = \left[\frac{A_{600\ nm\ control} - A_{600\ nm\ sample}}{A_{600\ nm\ control}}\right] \times 100$$
Chelating effect	The chelating effect was conducted according to the method of Lin et al. (2008). 1.0 mL of TLE solution (50 g of dry leaves L^{-1}) was mixed with 3.7 mL of methanol and 0.1 mL of 2 mM iron (II) chloride tetrahydrate for 30 seconds. Then, 0.2 mL of 5 mM ferrozine solution was added to the mixture. After 10 minutes, the absorbance of the reaction mixture was

	measured at 562 nm and compared to citric acid. $\text{Chelating Effect (\%)} = \left[\frac{A_{562\text{ nm of control}} - A_{562\text{ nm of sample}}}{A_{562\text{ nm of control}}}\right] \times 10$
Total polyphenol content	The TPC was conducted according to the method of Chen and Yen (2007). 0.2 mL of tree leaf extract solution (50 g of dry leaves L^{-1}) was mixed with 5.6 mL of DI water, 4 mL of 2% sodium carbonate, and 0.2 mL of 50% Folin & Ciocalteu's phenol reagent. The reaction was allowed to proceed for 30 minutes. Afterwards, the absorbance was measured at 750 nm. Standard calibration of gallic acid was conducted and the TPC was expressed as mg GAE/g of dried leaves. To obtain the value of dried samples, filtrate was placed in the oven at 50 ° C for 3 d and weighed.
Qualitative analysis of tree leaf polyphenol constituents - HPLC/PDA	For qualitative analysis of tree leaf polyphenol constituents, 1 mL of aqueous sample was withdrawn from each bottle of filtrate extract and filtered with polytetrafluorethylene (PTFE) filter (0.2 µm). Limited to the available standards, five phenolic standards were also prepared with a concentration of 500 mg L^{-1} namely caffeic acid, 4-hydrobenzoic acid, tannic acid, gallic acid, and vanillic acid. All the filtrate was analyzed using a high-performance liquid chromatograph (HPLC) (PerkinElmer Flexar Quaternary LC system) equipped with a photodiode array detector (PDA) with a Brownlee SPP C18 (4.6 × 1500 mm) column. The mobile phase was methanol and 1.5% acetic acid (25/75, v/v) at a flow rate of 0.8 mL min^{-1}, and the effluent was monitored by the PDA detector.
Qualitative analysis of tree leaf polyphenol constituents - FTIR-ATR	Fourier transform infrared spectroscopy-attenuated total reflectance (FTIR-ATR, Nicolet iS50 FTIR Spectrometer, Thermo Scientific) was employed to analyze the functional groups present in the tree leaf samples. Dried tree leaves were pulverized prior to the analysis. The wavenumber was set within the range of 4000-500 cm^{-1}, with 64 scans for every measurement at a resolution of 2 cm^{-1}.
Iron concentrations	Iron concentrations were determined using UV-Vis spectrophotometry with a HACH DR-3900 spectrophotometer. Total iron was measured using

	HACH Method 8008, and ferrous ion was measured using Method 8146. Ferric ion concentration was calculated by subtracting the ferrous ion content from the total iron.

References:
Chen, H.-Y., Yen, G.-C., 2007. Antioxidant activity and free radical-scavenging capacity of extracts from guava (Psidium guajava L.) leaves. Food Chemistry 101(2), 686-694. https://doi.org/10.1016/j.foodchem.2006.02.047.
Lin, S.-D., Liu, E.-H., Mau, J.-L., 2008. Effect of different brewing methods on antioxidant properties of steaming green tea. LWT - Food Science and Technology 41(9), 1616-1623. https://doi.org/10.1016/j.lwt.2007.10.009.
Yen, G.-C., Chen, H.-Y., 1995. Antioxidant activity of various tea extracts in relation to their antimutagenicity. Journal of Agricultural and Food Chemistry 43, 27-32. https://doi.org/10.1021/jf00049a007.

Table S4. The response value of each factor in the degradation of CT within the aqueous phase.

**Factor**	**Responses (Average CT Removal)**				**Responses (Average S/N Ratio)**			
	Level 1	Level 2	Level 3	Delta effect	Level 1	Level 2	Level 3	Delta effect
pH	63.63	87.90	94.40	24.78	36.00	38.85	39.50	3.50
Leaf dose (g/L water)	85.54	79.85	80.54	5.69	38.57	37.75	38.03	0.82
Iron dosage	80.04	79.91	85.99	6.09	38.01	37.75	38.59	0.83
